# Bis(μ-bis­{[4-(2-pyrid­yl)pyrimidin-2-yl]sulfan­yl}methane)­disilver(I) bis­(perchlorate)

**DOI:** 10.1107/S160053681004924X

**Published:** 2010-11-30

**Authors:** Hai-Bin Zhu

**Affiliations:** a School of Chemistry and Chemical Engineering, Southeast University, Nanjing 211189, People’s Republic of China

## Abstract

In the macrocyclic centrosymmetric dinuclear complex, [Ag_2_(C_19_H_14_N_6_S_2_)_2_](ClO_4_)_2_, the Ag^I^ atom, bis­{[4-(2-pyrid­yl)pyrimidin-2-yl]sulfan­yl}methane (2-bppt) ligand and perchlorate anion each lie on a twofold rotation axis. The 2-bppt ligand chelates two four-coordinated Ag^I^ atoms through its two bipyridine-like arms. The O atoms of the perchlorate anion are disordered each over two positions of equal occupancy. Adjacent complex mol­ecules are linked by π–π inter­actions between the pyridine and pyrimidine rings [centroid–centroid distance = 3.663 (8) Å].

## Related literature

For Ag(I) coordination polymers, see: Chen *et al.* (2006 ▶). For the coordination chemistry of 4-(pyridin-*n*-yl)pyrimidin-2-thiol (*n* = 2, 3, 4) and their derivatives, see: Dong *et al.* (2009 ▶); Huang *et al.* (2007 ▶); Zhu *et al.* (2010 ▶).
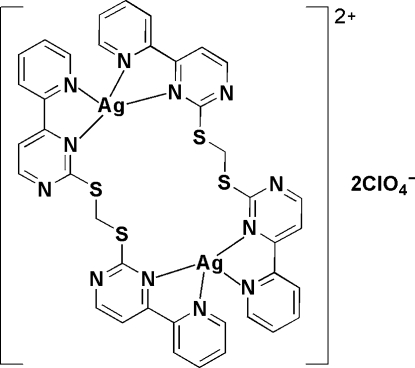


## Experimental

### 

#### Crystal data

[Ag_2_(C_19_H_14_N_6_S_2_)_2_](ClO_4_)_2_
*M* *_r_* = 1195.64
Orthorhombic, 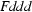

*a* = 10.4382 (16) Å
*b* = 27.896 (4) Å
*c* = 30.089 (5) Å
*V* = 8761 (2) Å^3^
*Z* = 8
Mo *K*α radiation
μ = 1.27 mm^−1^
*T* = 298 K
0.15 × 0.12 × 0.10 mm

#### Data collection

Bruker APEXII CCD diffractometer
Absorption correction: multi-scan (*SADABS*; Bruker, 2001 ▶) *T*_min_ = 0.832, *T*_max_ = 0.880
14503 measured reflections
2705 independent reflections
1640 reflections with *I* > 2σ(*I*)
*R*_int_ = 0.035

#### Refinement

*R*[*F* ^2^ > 2σ(*F* ^2^)] = 0.046
*wR*(*F* ^2^) = 0.172
*S* = 1.05
2705 reflections
169 parameters
24 restraints
H-atom parameters constrained
Δρ_max_ = 0.54 e Å^−3^
Δρ_min_ = −0.64 e Å^−3^

### 

Data collection: *APEX2* (Bruker, 2007 ▶); cell refinement: *SAINT-Plus* (Bruker, 2007 ▶); data reduction: *SAINT-Plus*; program(s) used to solve structure: *SHELXS97* (Sheldrick, 2008 ▶); program(s) used to refine structure: *SHELXL97* (Sheldrick, 2008 ▶); molecular graphics: *SHELXTL* (Sheldrick, 2008 ▶); software used to prepare material for publication: *SHELXTL*.

## Supplementary Material

Crystal structure: contains datablocks I, global. DOI: 10.1107/S160053681004924X/hy2383sup1.cif

Structure factors: contains datablocks I. DOI: 10.1107/S160053681004924X/hy2383Isup2.hkl

Additional supplementary materials:  crystallographic information; 3D view; checkCIF report

## Figures and Tables

**Table 1:** Selected bond lengths (Å)

Ag1—N1	2.277 (4)
Ag1—N2	2.398 (3)

**Fig. 1.**
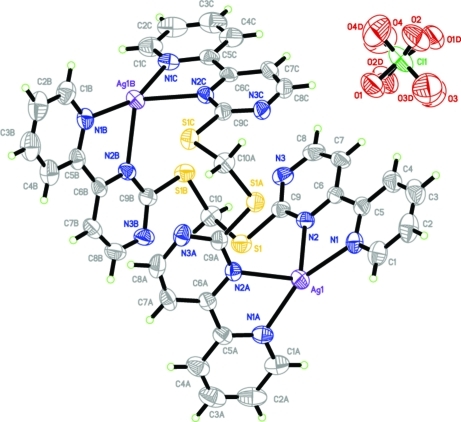
Molecular structure of the title compound, with the 30% probability displacement ellipsoids. [Symmetry codes: (A) 7/4 - x, 3/4 - y, z; (B) x, 3/4 - y, -1/4 - z; (C) 7/4 - x, y, -1/4 - z; (D) 9/4 - x, 1/4 - y, z.]

## supplementary crystallographic information

### Comment

The supramolecular chemistry of Ag(I) coordination polymers is being a dynamic and thriving research field, which has attracted considerable interest (Chen *et. al*, 2006). For a long time, we have focused on the coordination chemistry of 4-(pyridin-*n*-yl)pyrimidin-2-thiol (*n* = 2, 3, 4) and their derivatives (Dong *et al.*, 2009; Huang *et al.*, 2007; Zhu *et al.*, 2010). Herein, we report a macrocyclic Ag(I) complex with bis[4-(2-pyridyl)pyrimidin-2-ylthio]methane (2-bppt) ligand.

The title compound shows a discrete macrocylic dinuclear structure, with perchlorate anions uncoordinated (Fig. 1). Each Ag^I^ ion is chelated by two sets of N,*N*-chelating donors from two 2-bppt ligands. The Ag—N bond distances are 2.277 (4) and 2.398 (3) Å (Table 1), while the N—Ag—N angles are in the range of 70.96 (13) to 158.7 (2)°. The Ag—Ag separation across the macrocycle is 8.167 (1) Å.

### Experimental

A CH_3_CN solution of AgClO_4_ (0.1 mmol) was layered above a CH_2_Cl_2_ solution of 2-bppt (0.1 mmol). Colorless crystals were obtained after one week. The crystals were collected and dried under vacuum (yield: 46%).

### Refinement

All H atoms were positioned geometrically and allowed to ride on their parent atoms, with C—H = 0.93 and 0.97 Å and with *U*_iso_(H) = 1.2*U*_eq_(C).

### Crystal data

**Table:**

[Ag_2_(C_19_H_14_N_6_S_2_)_2_](ClO_4_)_2_	*F*(000) = 4768
*M**_r_* = 1195.64	*D*_x_ = 1.813 Mg m^−^^3^
Orthorhombic, *F**d**d**d*	Mo *K*α radiation, λ = 0.71073 Å
Hall symbol: -F 2uv 2vw	Cell parameters from 2705 reflections
*a* = 10.4382 (16) Å	θ = 2.3–25.5°
*b* = 27.896 (4) Å	µ = 1.27 mm^−^^1^
*c* = 30.089 (5) Å	*T* = 298 K
*V* = 8761 (2) Å^3^	Block, colorless
*Z* = 8	0.15 × 0.12 × 0.10 mm

### Data collection

**Table:**

Bruker APEXII CCD diffractometer	2705 independent reflections
Radiation source: fine-focus sealed tube	1640 reflections with *I* > 2σ(*I*)
graphite	*R*_int_ = 0.035
φ and ω scans	θ_max_ = 28.3°, θ_min_ = 2.2°
Absorption correction: multi-scan (*SADABS*; Bruker, 2001)	*h* = −13→12
*T*_min_ = 0.832, *T*_max_ = 0.880	*k* = −36→35
14503 measured reflections	*l* = −35→40

### Refinement

**Table:**

Refinement on *F*^2^	Primary atom site location: structure-invariant direct methods
Least-squares matrix: full	Secondary atom site location: difference Fourier map
*R*[*F*^2^ > 2σ(*F*^2^)] = 0.046	Hydrogen site location: inferred from neighbouring sites
*wR*(*F*^2^) = 0.172	H-atom parameters constrained
*S* = 1.05	*w* = 1/[σ^2^(*F*_o_^2^) + (0.1*P*)^2^] where *P* = (*F*_o_^2^ + 2*F*_c_^2^)/3
2705 reflections	(Δ/σ)_max_ < 0.001
169 parameters	Δρ_max_ = 0.54 e Å^−^^3^
24 restraints	Δρ_min_ = −0.64 e Å^−^^3^

### Fractional atomic coordinates and isotropic or equivalent isotropic displacement parameters (Å^2^)

**Table:**

	*x*	*y*	*z*	*U*_iso_*/*U*_eq_	Occ. (<1)
Ag1	0.8750	0.3750	0.010711 (18)	0.0724 (3)	
S1	0.64901 (13)	0.37857 (5)	−0.07449 (5)	0.0703 (4)	
Cl1	1.1250	0.1250	−0.08929 (11)	0.1119 (9)	
C6	0.8563 (4)	0.27205 (15)	−0.03645 (15)	0.0602 (11)	
N2	0.7995 (3)	0.31493 (12)	−0.03991 (12)	0.0577 (9)	
N3	0.6715 (4)	0.28754 (15)	−0.10059 (13)	0.0708 (10)	
C9	0.7124 (4)	0.32014 (15)	−0.07181 (14)	0.0598 (10)	
C5	0.9565 (5)	0.26651 (16)	−0.00209 (16)	0.0652 (12)	
C10	0.5575 (6)	0.3750	−0.1250	0.0715 (18)	
H10A	0.5025	0.4030	−0.1267	0.086*	0.50
H10B	0.5025	0.3470	−0.1233	0.086*	0.50
N1	0.9792 (4)	0.30490 (14)	0.02472 (13)	0.0693 (10)	
C8	0.7272 (6)	0.24468 (18)	−0.09552 (17)	0.0804 (15)	
H8	0.7013	0.2199	−0.1141	0.096*	
C7	0.8198 (6)	0.23519 (16)	−0.06458 (18)	0.0761 (14)	
H7	0.8571	0.2050	−0.0625	0.091*	
C3	1.1171 (7)	0.2208 (3)	0.0336 (3)	0.107 (2)	
H3	1.1630	0.1924	0.0365	0.128*	
C1	1.0704 (5)	0.3010 (2)	0.05571 (19)	0.0873 (15)	
H1	1.0864	0.3268	0.0745	0.105*	
C2	1.1432 (5)	0.2585 (3)	0.0605 (3)	0.105 (2)	
H2	1.2078	0.2565	0.0817	0.125*	
C4	1.0244 (6)	0.2245 (2)	0.0026 (2)	0.0892 (16)	
H4	1.0062	0.1985	−0.0158	0.107*	
O1	1.1499 (10)	0.1740 (3)	−0.0861 (3)	0.120 (3)	0.50
O2	1.2437 (12)	0.1043 (4)	−0.0898 (4)	0.142 (4)	0.50
O3	1.0656 (13)	0.1194 (5)	−0.0423 (5)	0.166 (5)	0.50
O4	1.0504 (13)	0.1200 (6)	−0.1265 (5)	0.176 (5)	0.50

### Atomic displacement parameters (Å^2^)

**Table:**

	*U*^11^	*U*^22^	*U*^33^	*U*^12^	*U*^13^	*U*^23^
Ag1	0.0848 (4)	0.0529 (3)	0.0795 (4)	−0.0060 (2)	0.000	0.000
S1	0.0718 (8)	0.0712 (7)	0.0679 (8)	0.0074 (5)	−0.0057 (6)	−0.0091 (6)
Cl1	0.1036 (17)	0.0619 (12)	0.170 (3)	0.0054 (11)	0.000	0.000
C6	0.068 (3)	0.051 (2)	0.062 (3)	−0.0062 (19)	0.026 (2)	0.0064 (19)
N2	0.059 (2)	0.0522 (18)	0.062 (2)	−0.0070 (15)	0.0100 (18)	−0.0039 (15)
N3	0.074 (2)	0.071 (3)	0.067 (2)	−0.022 (2)	0.007 (2)	−0.0131 (19)
C9	0.058 (2)	0.063 (2)	0.059 (2)	−0.0073 (19)	0.012 (2)	−0.006 (2)
C5	0.065 (3)	0.062 (3)	0.069 (3)	0.002 (2)	0.023 (2)	0.013 (2)
C10	0.050 (3)	0.089 (5)	0.076 (4)	0.000	0.000	0.000 (3)
N1	0.066 (2)	0.068 (2)	0.074 (2)	−0.0072 (19)	0.004 (2)	0.0169 (19)
C8	0.095 (4)	0.075 (3)	0.071 (3)	−0.026 (3)	0.020 (3)	−0.020 (3)
C7	0.091 (4)	0.050 (2)	0.087 (4)	−0.008 (2)	0.034 (3)	−0.007 (2)
C3	0.092 (5)	0.102 (5)	0.126 (6)	0.029 (4)	0.023 (4)	0.036 (5)
C1	0.074 (3)	0.101 (4)	0.087 (4)	−0.006 (3)	−0.007 (3)	0.023 (3)
C2	0.065 (4)	0.129 (6)	0.120 (5)	0.007 (3)	−0.003 (3)	0.050 (5)
C4	0.087 (4)	0.081 (3)	0.099 (4)	0.022 (3)	0.027 (3)	0.019 (3)
O1	0.152 (7)	0.076 (5)	0.132 (7)	0.008 (5)	−0.029 (5)	−0.009 (4)
O2	0.133 (7)	0.105 (6)	0.188 (8)	0.044 (6)	−0.017 (6)	−0.012 (6)
O3	0.176 (9)	0.166 (8)	0.155 (8)	−0.016 (7)	0.028 (7)	0.007 (7)
O4	0.167 (8)	0.198 (9)	0.162 (8)	−0.025 (8)	−0.076 (7)	−0.041 (7)

### Geometric parameters (Å, °)

**Table:**

Ag1—N1	2.277 (4)	C5—C4	1.377 (6)
Ag1—N2	2.398 (3)	C10—S1^i^	1.798 (4)
S1—C9	1.761 (4)	C10—H10A	0.9700
S1—C10	1.798 (4)	C10—H10B	0.9700
Cl1—O4	1.370 (11)	N1—C1	1.337 (6)
Cl1—O2	1.367 (11)	C8—C7	1.368 (8)
Cl1—O1	1.395 (9)	C8—H8	0.9300
Cl1—O3	1.552 (13)	C7—H7	0.9300
C6—N2	1.339 (5)	C3—C2	1.355 (11)
C6—C7	1.385 (7)	C3—C4	1.348 (9)
C6—C5	1.479 (7)	C3—H3	0.9300
N2—C9	1.330 (5)	C1—C2	1.416 (9)
N3—C9	1.326 (5)	C1—H1	0.9300
N3—C8	1.338 (6)	C2—H2	0.9300
C5—N1	1.361 (6)	C4—H4	0.9300
			
N1—Ag1—N1^ii^	158.7 (2)	S1^i^—C10—H10A	108.3
N1—Ag1—N2	71.00 (14)	S1—C10—H10B	108.3
N1^ii^—Ag1—N2	124.11 (13)	S1^i^—C10—H10B	108.3
N1—Ag1—N2^ii^	124.11 (13)	H10A—C10—H10B	107.4
N1^ii^—Ag1—N2^ii^	71.00 (14)	C5—N1—C1	118.3 (5)
N2—Ag1—N2^ii^	101.13 (16)	C5—N1—Ag1	118.9 (3)
C9—S1—C10	100.78 (17)	C1—N1—Ag1	122.6 (4)
O4—Cl1—O4^iii^	70.5 (13)	N3—C8—C7	123.9 (4)
O4—Cl1—O2	117.6 (10)	N3—C8—H8	118.1
O4^iii^—Cl1—O2	61.2 (7)	C7—C8—H8	118.1
O4—Cl1—O2^iii^	61.2 (7)	C8—C7—C6	117.8 (5)
O4^iii^—Cl1—O2^iii^	117.6 (10)	C8—C7—H7	121.1
O2—Cl1—O2^iii^	178.7 (11)	C6—C7—H7	121.1
O4—Cl1—O1^iii^	81.5 (8)	C2—C3—C4	119.8 (6)
O4^iii^—Cl1—O1^iii^	105.2 (8)	C2—C3—H3	120.1
O2—Cl1—O1^iii^	75.8 (6)	C4—C3—H3	120.1
O2^iii^—Cl1—O1^iii^	104.3 (6)	N1—C1—C2	121.3 (6)
O4—Cl1—O1	105.2 (8)	N1—C1—H1	119.3
O4^iii^—Cl1—O1	81.5 (8)	C2—C1—H1	119.3
O2—Cl1—O1	104.3 (6)	C3—C2—C1	118.9 (6)
O2^iii^—Cl1—O1	75.8 (6)	C3—C2—H2	120.6
O1^iii^—Cl1—O1	172.0 (9)	C1—C2—H2	120.6
O4—Cl1—O3^iii^	168.9 (8)	C3—C4—C5	120.3 (6)
O4^iii^—Cl1—O3^iii^	120.5 (8)	C3—C4—H4	119.8
O2—Cl1—O3^iii^	72.0 (7)	C5—C4—H4	119.8
O2^iii^—Cl1—O3^iii^	109.3 (9)	Cl1—O1—O2^iii^	51.3 (4)
O1^iii^—Cl1—O3^iii^	96.3 (7)	Cl1—O1—O4^iii^	48.6 (5)
O1—Cl1—O3^iii^	76.3 (7)	O2^iii^—O1—O4^iii^	83.8 (7)
O4—Cl1—O3	120.5 (8)	Cl1—O1—O3^iii^	55.7 (6)
O4^iii^—Cl1—O3	168.9 (8)	O2^iii^—O1—O3^iii^	85.0 (7)
O2—Cl1—O3	109.3 (9)	O4^iii^—O1—O3^iii^	88.7 (8)
O2^iii^—Cl1—O3	72.0 (7)	Cl1—O2—O4^iii^	59.5 (7)
O1^iii^—Cl1—O3	76.3 (7)	Cl1—O2—O1^iii^	52.8 (5)
O1—Cl1—O3	96.3 (7)	O4^iii^—O2—O1^iii^	90.0 (9)
O3^iii^—Cl1—O3	48.6 (10)	Cl1—O2—O3^iii^	59.0 (6)
N2—C6—C7	119.6 (5)	O4^iii^—O2—O3^iii^	108.6 (10)
N2—C6—C5	117.4 (4)	O1^iii^—O2—O3^iii^	80.0 (8)
C7—C6—C5	123.0 (4)	O3^iii^—O3—Cl1	65.7 (5)
C9—N2—C6	117.2 (4)	O3^iii^—O3—O2^iii^	104.2 (9)
C9—N2—Ag1	127.3 (3)	Cl1—O3—O2^iii^	49.0 (5)
C6—N2—Ag1	115.4 (3)	O3^iii^—O3—O1^iii^	88.3 (12)
C9—N3—C8	113.5 (4)	Cl1—O3—O1^iii^	48.0 (5)
N3—C9—N2	128.0 (4)	O2^iii^—O3—O1^iii^	75.8 (7)
N3—C9—S1	119.0 (4)	Cl1—O4—O2^iii^	59.3 (7)
N2—C9—S1	113.0 (3)	Cl1—O4—O4^iii^	54.8 (6)
N1—C5—C4	121.3 (5)	O2^iii^—O4—O4^iii^	103.6 (10)
N1—C5—C6	117.1 (4)	Cl1—O4—O1^iii^	49.9 (6)
C4—C5—C6	121.6 (5)	O2^iii^—O4—O1^iii^	84.9 (9)
S1—C10—S1^i^	115.8 (4)	O4^iii^—O4—O1^iii^	80.6 (11)
S1—C10—H10A	108.3		

Symmetry codes: (i) *x*, −*y*+3/4, −*z*−1/4; (ii) −*x*+7/4, −*y*+3/4, *z*; (iii) −*x*+9/4, −*y*+1/4, *z*.
